# Neonatal cardiac hypertrophy: the role of hyperinsulinism—a review of literature

**DOI:** 10.1007/s00431-019-03521-6

**Published:** 2019-12-16

**Authors:** Nina D. Paauw, Raymond Stegeman, Monique A. M. J. de Vroede, Jacqueline U. M. Termote, Matthias W. Freund, Johannes M. P. J. Breur

**Affiliations:** 1 Department of Obstetrics, Wilhelmina Children’s Hospital Birth Center, University Medical Center Utrecht, Utrecht, The Netherlands; 2 Department of Pediatric Cardiology, Wilhelmina Children’s Hospital, University Medical Center Utrecht, PO Box 85090, 3508 AB Utrecht, The Netherlands; 3 Department of Pediatric Endocrinology, Wilhelmina Children’s Hospital, University Medical Center Utrecht, Utrecht, The Netherlands; 4 Department of Neonatology, Wilhelmina Children’s Hospital Birth Center, University Medical Center Utrecht, Utrecht, The Netherlands; 5 Department of Pediatric Cardiology, Klinikum Oldenburg, University of Oldenburg, Oldenburg, Germany

**Keywords:** Hypertrophic cardiomyopathy, Cardiac hypertrophy, Hyperinsulinemia, Hyperinsulinemic state

## Abstract

Hypertrophic cardiomyopathy (HCM) in neonates is a rare and heterogeneous disorder which is characterized by hypertrophy of heart with histological and functional disruption of the myocardial structure/composition. The prognosis of HCM depends on the underlying diagnosis. In this review, we emphasize the importance to consider hyperinsulinism in the differential diagnosis of HCM, as hyperinsulinism is widely associated with cardiac hypertrophy (CH) which cannot be distinguished from HCM on echocardiographic examination. We supply an overview of the incidence and treatment strategies of neonatal CH in a broad spectrum of hyperinsulinemic diseases. Reviewing the literature, we found that CH is reported in 13 to 44% of infants of diabetic mothers, in approximately 40% of infants with congenital hyperinsulinism, in 61% of infants with leprechaunism and in 48 to 61% of the patients with congenital generalized lipodystrophy. The correct diagnosis is of importance since there is a large variation in prognoses and there are various strategies to treat CH in hyperinsulinemic diseases.

*Conclusion*: The relationship between CH and hyperinsulism has implications for clinical practice as it might help to establish the correct diagnosis in neonates with cardiac hypertrophy which has both prognostic and therapeutic consequences. In addition, CH should be recognized as a potential comorbidity which might necessitate treatment in all neonates with known hyperinsulinism.

**Table:** 

**What is Known:** • *Hyperinsulinism is currently not acknowledged as a cause of hypertrophic cardiomyopathy (HCM) in textbooks and recent Pediatric Cardiomyopathy Registry publications.* **What is New:** • *This article presents an overview of the literature of hyperinsulinism in neonates and infants showing that hyperinsulinism is associated with cardiac hypertrophy (CH) in a broad range of hyperinsulinemic diseases.* • *As CH cannot be distinguished from HCM on echocardiographic examination, we emphasize the importance to consider hyperinsulinism in the differential diagnosis of HCM/CH as establishing the correct diagnosis has both prognostic and therapeutic consequences.*

**What is Known:**

• *Hyperinsulinism is currently not acknowledged as a cause of hypertrophic cardiomyopathy (HCM) in textbooks and recent Pediatric Cardiomyopathy Registry publications.*

**What is New:**

• *This article presents an overview of the literature of hyperinsulinism in neonates and infants showing that hyperinsulinism is associated with cardiac hypertrophy (CH) in a broad range of hyperinsulinemic diseases.*

• *As CH cannot be distinguished from HCM on echocardiographic examination, we emphasize the importance to consider hyperinsulinism in the differential diagnosis of HCM/CH as establishing the correct diagnosis has both prognostic and therapeutic consequences.*

## Introduction

Hypertrophic cardiomyopathy (HCM) is defined as a disease in which the heart muscle becomes abnormally thick in the absence of abnormal loading conditions (hypertension, valve disease) with histological disruption of the myocardial structure/composition and in the absence of systemic disease [1]. In adults, HCM has a prevalence of 0.2% and a genetic origin in most of the cases. In contrast, childhood HCM is rare with a reported mean incidence of 4.7 per million, with a highest incidence in children < 1 year is found (30 per million) [2]. In children, HCM has a heterogeneous etiology and is more often reported secondary to conditions such as endocrine and metabolic disorders. An extensive table of etiologies of pediatric HCM is listed in the excellent review by Moak et al. [3]. Inborn errors of metabolism (storage disease and mitochondrial disorders), malformation syndromes, and neuromuscular disorders contribute to one third of the pediatric HCM, each reported in around 10% of cases of pediatric CHM. Sacromeric protein mutations resulting in HCM are found in about 50% of the cases; however, in the other half, a specific cause cannot be identified (idiopathic HCM) [3–5].

Hyperinsulinism is not generally mentioned as a cause of HCM in textbooks [6] and recent Pediatric Cardiomyopathy Registry publications [7], while a relation between hyperinsulinism and hypertrophy of the heart is widely reported in relation to hyperinsulinism such as in neonates of diabetic mothers in 1980 [8, 9] in patients with congenital hyperinsulinism (CHI) [10], as well as in patients with leprechaunism [11] and congenital lipodystrophy [12]. This absence of hyperinsulinism in the list of differential diagnosis of HCM is probably due to the fact that hyperinsulinism does not per se cause HCM but is associated with cardiac hypertrophy (CH) without meeting the criteria of histological and functional disruption needed to diagnose HCM. Despite this, CH is often, incorrectly, termed HCM in current literature. This might be caused by the fact that the distinction between HCM and CH cannot be made completely using echocardiographic evaluation. Therefore, we think that in the case of thickening of the heart muscle on ultrasound which suggests the presence of HCM, CH due to hyperinsulinemia should also be considered in the differential diagnosis, especially since the diagnosis might have consequences for the prognosis and treatment of the neonate.

In order to highlight the importance of the association of hyperinsulinemia and CH, this article presents a review on the literature of hyperinsulinism in neonates and infants to determine the frequency of CH in this specific population. In addition, we will discuss by which mechanisms CH might develop during hyperinsulinemic states and in which ways the diagnosis of hyperinsulinemic CH affects further work-up and treatment. A literature search was performed in the PubMed electronic database using the search terms: neonate and infant, in combination with hyperinsulinism and hypertrophic cardiomyopathy or cardiac hypertrophy. Appropriate articles were selected by two reviewers (NP and RS). Snowballing was performed using references cited in publications retrieved during the database search. In this report, we will strictly use the term HCM for the genetic variants that cause sarcomeric hear disease and CH for all other kinds of hypertrophy of the heart muscle. The term CH indicates the presence of left ventricular or septal hypertrophy following the definition of echocardiographic measured diastolic septal thickness or diastolic left ventricular wall thickness ≥ 2 standard deviations above the mean (Z-score ≥ 1.96; corrected for age, sex, and body size) [13–15].

## Association between cardiac hypertrophy and hyperinsulinism

Our search revealed that the association between CH and hyperinsulinism is mainly observed in the neonate, although the occurrence in older infants has also been described [16, 17]. The etiology of hyperinsulinism in the neonate and infant can be subdivided into three categories: maternal diabetes, congenital hyperinsulinism (transient and persistent), and insulin resistance syndromes. Additionally, hyperinsulinism is sometimes observed in syndromes in which the mechanism behind the development of hyperinsulinism remains to be resolved such as in Costello syndrome. Syndromes such as Noonan and Leopard in which CH is often reported will not be included since hyperinsulinism is not a typical feature.

### Maternal diabetes

Hyperglycemia in maternal diabetes can lead to fetal hyperinsulinism which persists transiently in the neonatal period. Infants of diabetic mothers may present with a variety of metabolic problems and have an increased risk of congenital malformations. As for the heart, patent ductus arteriosus and CH are the most prevalent abnormalities [18–21]*.* The exact incidence of CH in children of diabetic mothers remains unclear since the CH is asymptomatic in most cases. A literature search revealed nine studies reporting the numbers of CH in neonates of diabetic mothers (Table 1). The incidence ranged from 13 to 44% in mixed groups of asymptomatic and symptomatic neonates [9, 24, 26]. The widespread incidence of CH probably results from the lack of uniformity in these studies in terms of patient selection (type 1, type 2, and gestational diabetes), the degree of maternal glucose control, and the moment and place of referral. None of the studies reported the incidence of CH in small for gestational age babies or in neonates of diabetic mothers with worse glucose control.

**Table 1:** Incidences of cardiac hypertrophy in infants of diabetic mothers

Study	Type of diabetes	Population characteristics	Moment 1st ultrasound	Patients (number)	CH (%)
Gutgesell (1980) [9]	DM1, DM2, GD	Symptomatic, asymptomatic neonates	≤ 1st week postn	47	32
Sheehan (1986) [21]	N/R	Well-controlled diabetes	N/R	20	35
Deorari (1989) [22]	DM1,2 (16%), GD (84%)	High risk antenatal clinic	≤ 5 days postn	31	26
Vural (1995) [23]	N/R	NICU or neonatology ward	N/R	56	30
Oberhoffer (1997) [24]	DM1 (59%), GD (41%)	Tightly controlled diabetic pregnancies	≤ 5 days postn	104	25
Abu-Sulaiman (2004) [18]	Insulin dependent DM	Tertiary care centrum	≤ 48 h postn	100	38
Tan (2005) [25]	DM1,2 (20%), GD (50%), IGT (30%)	Antenatal clinic	≤ 48 h postn	50	13
Ullmo (2006) [26]	DM1 (18%), DM2 (20%), GD (62%)	Perinatal care unit	Pren with post-FU	92	13
El-Ganzouriy (2012) [27]	DM1 (35%), DM2 (17%), GD (48%)	NICU	≤ 48 h postn	69	44
				569	28 (13–44)

*DM 1* diabetes mellitus type 1, *DM 2* diabetes mellitus type 2, *FU* follow-up, *GD* gestational diabetes, *HCM* hypertrophic cardiomyopathy, *IGT* impaired glucose tolerance, *N/R* not reported, *NICU* neonatal intensive care unit, *postn* postnatally, *pren* prenatally

A few studies showed a correlation between the development of CH in children and the type of maternal diabetes. Ullmo et al. showed that fetuses of mothers with type 1 diabetes have the highest risk to develop CH, followed by type 2 diabetes and only a low percentage in gestational diabetes [26]. In addition, children with CH had mothers with higher HbA1c levels compared to those of children without CH. In line with this, El-Ganzoury et al. found that higher HbA1c values were associated with a hypertrophied interventricular septum [27]. Several studies showed a trend towards increased CH in neonates with high birth weight [18, 27].

The effects of strict glycemic control to prevent CH remains controversial. Presumably in some studies the incidence of complications diminished by a rigorous control of maternal glycemia [26]. Other studies show that fetal CH may occur even with good glycemic control [21, 24]. To prevent CH, glucose control might even need to be more stringent than current recommendations. However, it cannot be ruled out that other unknown factors may be involved in the development of CH.

CH in neonates of mothers with diabetes is reversible as the stimulus for the insulin production disappears and is in most situations no longer detected on ultrasound after 6 months postnatally [24]. However, studies on the long-term cardiovascular consequences of transient cardiac hypertrophy in infancy are lacking.

### Congenital hyperinsulinism

Congenital hyperinsulinism (CHI), previously called “nesiodioblastosis,” can both be transient and persistent. The latter is also known as persistent hyperinsulinemic hypoglycemia of infancy (PPHI). Transient hyperinsulinism is generally caused by stressful conditions, such as perinatal asphyxia or intra-uterine growth restriction [28]. The pathophysiologic mechanism is unknown, and the hyperinsulinism disappears spontaneously within days or weeks after birth. In two newborns with transient congenital hyperinsulinism, severe obstructive but reversible CH was reported [29] (Table 2a). In persistent congenital hyperinsulinism, there is a focal or diffuse overproduction of insulin by the pancreas secondary to various genetic disorders. The incidence is estimated at 1 in 50,000 live births [34]. Hypoglycemia is the main feature and is associated with a high risk of seizures and cerebral morbidity. In most cases, persistent congenital hyperinsulinism is due to genetic defects in the pathway that regulate insulin secretion as outlined in a recent review [35]. Mutations in the Kir6.2 (KCNJ11 gene, omim #600937) and SUR1 (ABCC8 gene, omim #600509) subunits lead to permanent closure of the channel and account for 40–45% of all cases of congenital hyperinsulinism [36]. Until recently, CH was only reported in a few neonates with congenital hyperinsulinism [10, 30, 31]. In 2013, Huang et al. reviewed the charts of 68 infants with CHI. In 25 patients, an echocardiogram was performed of which 10 (40%) had CH and all of them required pancreatectomy [33]. After pancreatectomy, all patients showed improvement or complete resolution of the cardiac hypertrophy and accompanying cardiac dysfunction (Table 2b). The reported pattern of hypertrophy in these infants was mainly septal. Although CH in CHI is generally rather mild, it was occasionally reported to progress very rapidly to fatal obstructive CH .

**Table 2:** Cardiac hypertrophy in transient (A) and persistent (B) congenital hyperinsulinism (CHI)

Study	Population characteristics	Moment 1st ultrasound	Patients (number)	CH (%)	Treatment	Outcome
A transient CHI						
Mehta (2003) [29]	Atypical severe transient HOCM in CHI	N/R	2	^a^	Octreotide	Improvement 6W Complete resolution 3M
B Persistent CHI						
Harris (1992) [10]	Progressive CH in persistent CHI	N/R	1	^a^	Pancreatectomy	Condition resolved
Massin (1999) [30]	Progressive CH in focal CHI	N/R	1	^a^	Pancreatectomy	Complete resolution
Natarajan (2007) [31]	CH in CHI ABCC8 mutation	D10	1	^a^	Diazoxide, octreotride	Regressed spontaneously 8M
Zerah (2013) [32]	Rapidly progressive CH in CHI	D12/13	2	^a^	Diazoxide	Died
Huang (2013) [33]	CH in CHI ABCC8 (*n* = 7), KCNJ11 (*n* = 1) mutation	Early postn	25	40	Pancreatectomy	Improved/resolved

*CHI* congenital hyperinsulinism, *D* day, *HCM* hypertrophic cardiomyopathy, *HOCM* hypertrophic obstructive cardiomyopathy, *N/R* not reported, *M* months, *Postn* postnatally, *W* weeks

^a^Not reported, since all patients with CHI (100%) in these case reports showed HCM

### Other causes of persistent hyperinsulinemic hypoglycemia of infancy

There are no reports of CH in other PHHI syndromes such as hyperinsulinism-hyperammonemia (HI/HA) syndrome (omim #606762) and in hyperinsulinemic hypoglycemia due to an activating mutation in the glucokinase gene (omim #602485). Both syndromes are less common compared to K-ATP CHI. The lack of reports of CH in these syndromes might result from the lower prevalence of these diseases or their less aggressive course.

### Insulin resistance syndromes

Insulin resistance in the neonatal period and early life is associated with a few rare (mono)genetic syndromes. The main characteristics of insulin resistance syndromes are hyperglycemia, despite hyperinsulinemia, acanthosis nigricans, and gonadal dysfunction. CH is reported in a few patients with insulin resistance syndromes. The incidence of CH in the various forms of insulin resistance syndromes will be further outlined below.

### Leprechaunism (Donohue syndrome omim #246200)

Leprechaunism is a rare disease with an incidence of less than 1 per million births. The disorder is autosomal recessively inherited and characterized by extreme insulin resistance due to mutations in the insulin receptor (INSR) gene [37]. Features of the disease are severe intra-uterine growth retardation, diminished fat and muscle tissue, characteristic facies, and precocious puberty [38]. Termote et al. reviewed the literature of leprechaunism between 1980 and 2014 and retrieved 81 cases of leprechaunism of which 39 patients underwent cardiac examination [39]. Sixty-one percent of these patients showed signs of CH (Table 3). Mortality is extremely high in patients with leprechaunism [42–44] and 85% of the patients with CH die from heart failure [39]. The reported pattern of hypertrophy is mainly septal causing obstruction of the aortic outflow tract. In addition, a few authors report left- or biventricular hypertrophy [11, 43].

**Table 3:** Cardiac hypertrophy in insulin resistance syndromes

Study	Insulin resistance syndrome	Population characteristics	Moment 1st ultrasound	Patients (number)	CH (%)	Treatment	Outcome
Termote (2016) [39]	Leprechaunism (Donohue syndrome)∗	HOCM	21D	1 39‡	† 61‡	Metformin	Died 2.5M
Friguls (2009) [17]	BSCL§	HOCM	4M	1	†	Dietary	Normalized 2Y
Lupsa (2010) [40]	Congenital generalized lipodystrophy	AGPAT2 Seipin LMNA Unknown		31 − 19 − 10 − 1 − 1	61 − 53 − 80 − 100 − 0		Severe CHF (*n* = 2) Died (*n* = 4) by CHF
Jeninga (2012) [41]	BSCL††	HOCM	4M	1	†	Dietary, insulin, metformin	Normalized 16M

*HOCM* Hypertrophic obstructive cardiomyopathy, *BSCL* Berardinelli-Seip congenital lipodystrophy, *CHF* chronic hard failure

### Congenital generalized lipodystrophy

Congenital generalized lipodystrophy includes a group of genetic diseases characterized by disturbances of adipocytes causing low body fat, accumulation of lipid in muscle and liver, and insulin resistance resulting in hyperinsulinemia [45]. Less than 300 cases are reported to date and mutations in several loci are found of which the most relevant are AGPAT2 in congenital generalized lipodystrophy type 1 (omim #608594), seipin in congenital generalized lipodystrophy type 2 (BSCL2) (omim #269700), caveolin 1 in congenital generalized lipodystrophy type 3 (omim #612526), and PTRF in congenital generalized lipodystrophy type 4 (omim #613327) [46]. The proteins encoded by these genes have a key role in the lipid synthesis and droplet formation in the adipose tissue. Caveolin and PTRF are involved in caveolae formation, which are plasma membrane invaginations in which free fatty acids are taken up and conversed to triacylglycerols. AGPAT2 is the key enzyme in the synthesis of triacylglycerols and glycerophospholipids. The role of seipin is less clear, but it may be involved in the formation of lipid droplets [47]. CH is a feature that is observed in 48 to 61% of the patients with congenital generalized lipodystrophy [40] and has a higher prevalence in patients with seipin mutations (80%) than with AGPAT2 (53%), caveolin 1, and PTRF mutations [48] (Table 3). Although CH generally develops in the third decade, occasionally CH is observed before the age of 1 year [16, 17, 47]. The CH pattern in lipodystrophy patients is asymmetrical [16, 49, 50].

### Other hyperinsulinemic syndromes with insulin resistance

In patients with Rabson Mendenhal (omim #262190) and Type A syndrome (omim #610549), which are rare insulin resistance syndromes, CH is not reported in literature. This might be due to the fact that these patients in general have a more benign course and are diagnosed later in life [44].

### Beckwith-Wiedemann (omim #130650)

Beckwith-Wiedemann (BWS) is a congenital overgrowth syndrome with a reported incidence of 1:13.700 births. BWS is a complex multigenic disorder caused by dysregulation of imprinted growth regulatory genes within the chromosome 11p15 region including increased activity of the *IGF-2* gene in many tissues [51]. Features of the disease are macroglossia, generalized visceromegaly, anterior abdominal wall defects, hemihypertrophy, an accelerated growth potential, and increased risk of tumor formation [52]. Hyperinsulinemic hypoglycemia is observed in 30–50% of the patients [53–55]. In the majority of the BWS/11P overgrowth patients, the hypoglycemia is asymptomatic and resolves spontaneously. Only 5% of the BWS patients have hypoglycemia beyond the neonatal period. This persistent and severe hyperinsulinism phenotype is the result of a paternal uniparental isodisomy for chromosome 11p (pUPD11p) [56]. Most of these patients are refractory to diazoxide treatment and about half of them require a subtotal pancreatectomy.

Cardiac involvement in BWS/11P overgrowth patients is mostly limited to mild spontaneously-resolving CH (Table 4). However, in patients with BWS and severe hyperinsulinism (by pUPD11p), it might be severe and obstructive [58]. The pattern of hypertrophy is mainly characterized by hypertrophy of the septal region [58, 64].

**Table 4:** Cardiac hypertrophy in hyperinsulinism-associated syndromes

Study	Hyperinsulinism-associated syndrome	Patients (number)	CH (%)	Treatment	Outcome
Greenwood (1977) [57]	BWS	13	*38*	N/R	N/R
Ryan (1989) [58]	BWS	1	^a^	Diazoxide	Resolution 7M
Zerah (2013) [115]^b^	BWS	1	^a^	Diazoxide	Died
Siwik (1998) [59]	Costello	30	*20*	N/R	N/R
Lin (2002) [60]	Costello	94	*34*	N/R	N/R
Dickson (2004) [61]	Costello	1	^a^	N/R	Died
Sheffield (2015) [62]	Costello	1	^a^	None	Died
Sun (2005) [63]	CDG	1	^a^	None	Died

*BWS* Beckwith-Wiedemann Syndrome, *CDG* Congenital Disorders of Glycosylation, *N/R* not reported

^a^Not reported, since all patients with CHI (100%) in these case reports showed HCM

^b^Data from abstract of Zerah et al. [32]

### Costello syndrome (omim #218040)

Costello syndrome is a rare congenital disease caused by mutations in the HRAS proto-oncogene [65]. Features of the disease are prenatally increased growth, postnatal growth retardation, coarse face, loose skin, developmental delay, and susceptibility for papillomata and malignancies [66]. Cardiac abnormalities are reported in 60–75% of the patients with Costello syndrome. These include a heterogeneous group of structural defects, cardiomyopathies, and tachyarrhytmias [59, 60]. CH is found in 20–34% of the Costello patients **(**Table 4). However, the relationship between CH and hyperinsulinism is unproved in this syndrome since increased insulin levels were not reported in most patients with CH. In only four patients with CH, hyperinsulinemic hypoglycemia was described [61, 62, 67]. Interestingly, the histology of the heart was reported to be similar to the cardiac hypertrophy seen in infants of diabetic mothers [61].

### Congenital disorders of glycosylation

Congenital disorders of glycosylation (CDG) are a genetically heterogeneous group of autosomal recessive disorders caused by defective biosynthesis or transfer of lipid-linked oligosaccharides or compromised processing of protein-bound oligosaccharides [68]. The clinical spectrum of the different CDG varies from multisystem to organ-restricted disease. Among the many CDG subtypes, CDG I (a,b,d) patients may suffer from hyperinsulinemic hypoglycemia [63, 69–71]. CH in these patients is rare, but it can be severe and obstructive [72]. The relationship of CH with hyperinsulinism is weak, since only one patient out of the reported cases of CH had hyperinsulinism (Table 4).

### Prenatal diagnosed CH

Occasionally CH is diagnosed prenatally [73]. One study reports that 7 out of 33 fetuses (21%) with prenatally diagnosed CH were related to maternal diabetes [74]. In this study, no other hyperinsulinemic causes were mentioned and the remaining prenatally diagnosed CH cases were associated with Noonan’s syndrome, alpha-thalassemia, twin-twin transfusion syndrome, and familial hypertrophy. Another study showed that the septal thickness in fetuses of diabetic mothers with CH is significantly associated with fetal insulin levels [75]. In addition, a follow-up study of the same group reports an association between regression of ventricular septum thickness and the decrease of insulin levels in the postpartum period in these prenatally diagnosed babies [76]. Prenatally diagnosed CH was not reported in one of the other mentioned hyperinsulinemic states.

### Morphologic, histologic, and hemodynamic characteristics of CH in hyperinsulinemic infants

**Fig. 1:**
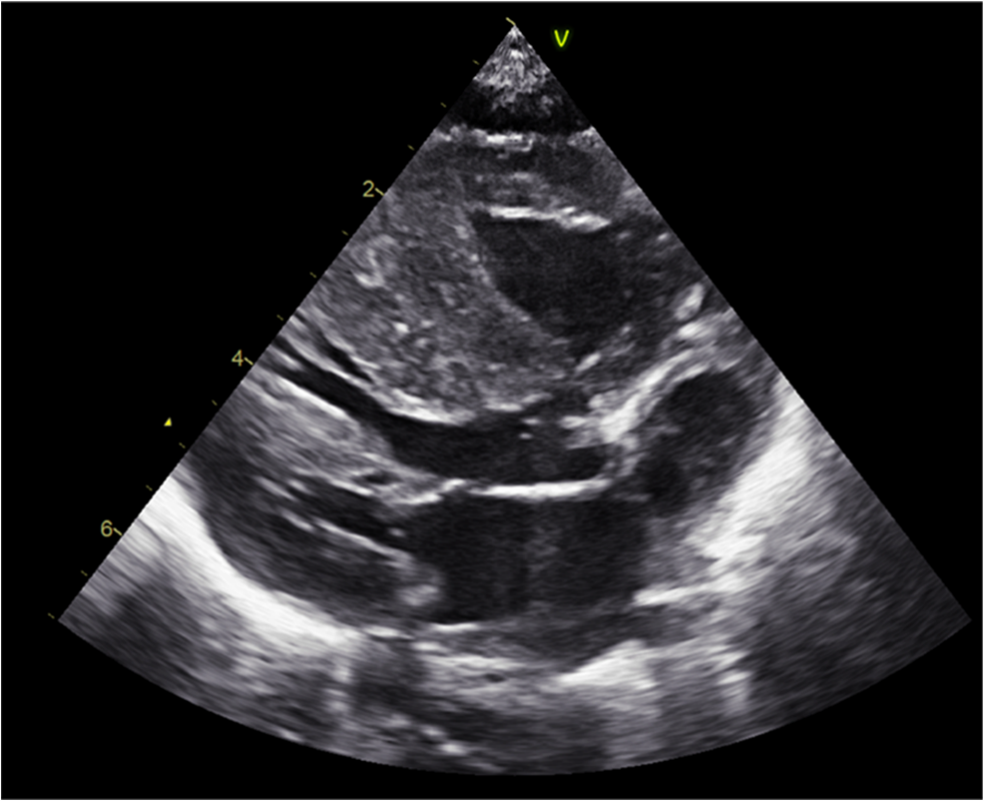
Echocardiographic images taken from a patient with congenital hyperinsulinism. The ultrasound features of the heart of patient with insulin-associated cardiac hypertrophy show specific thickening of the intraventricular septum

The pattern of CH related to hyperinsulinemic states is often described septal hypertrophy or left ventricular outflow tract obstruction [9, 77] (Fig. 1). On cardiac ultrasound, hearts of children with hyperinsulinemic disease are difficult to distinguish from other causes of CH as septal hypertrophy is the most common pattern described in HCM caused by sacromeric mutations. However, CH in the context of hyperinsulinism seems to differ microscopically and hemodynamically from other causes of CH. While CH in context of hyperinsulinism is characterized by diffuse hyperplasia of cardiomyocytes with normal configuration, histologic examination of non-hyperinsulinemic CH is typically characterized by disarray of the muscle fibers [78], intramyocardial fibrosis [79], and interstitial collagen depositions [80]. These findings suggest that hyperinsulinemic hypertrophy is an exaggeration of normal cardiac growth due to overstimulation by growth factors affecting the heart as a whole rather than an intrinsic disease of the heart muscle. This might also explain the differences in pathophysiologic processes leading to cardiac symptoms and death. In non-hyperinsulinemic CH patients, myocyte disarray and fibrosis lead to electric instability causing dysarrhytmias and sudden death. However, in hyperinsulinemic patients, it is frequently observed that CH results in a stiff heart with high diastolic filling pressure resulting in reduced stroke volume and in the case of severe CH leads to the development of heart failure secondary to left ventricular outflow tract obstruction [58, 81]. In most of the hyperinsulinemic patients, the hypertrophy is asymptomatic and even reversible after normalization of insulin levels [24, 30, 41, 82].

## Insulin as a cardiac growth factor?

The observed CH in hyperinsulinemic neonates and infants is presumably an exaggeration of the normal prenatal cardiac growth, in which biochemical signals and hormones including thyroid hormone, insulin, growth hormone, and IGF-I are involved [83]. The IGF/insulin system is composed by three ligands (IGF-I, IGF-II, and insulin), four receptors (insulin, IGF-I, IGF-II, and hybrid insulin/IGF-I), and six IGF-binding proteins that regulate the bioactivity of the IGFs. Besides the insulin and IGF-I receptor, hybrid Ins/IGF receptors are described, that can function as IGF-I receptors and will not be further discussed. IGF-II, which is also highly homologous with IGF-I binds to the IGF-II receptor, which has as an intracellular IGF-II clearance function as well as to the insulin and IGF-I receptor and mainly has a role in prenatal development. Insulin and IGF-1 are polypeptides with a high degree of homology and interact with each other receptors [84]. Below, we discuss how both insulin and IGF1 signaling with their receptors might act as a cardiac growth factor. Although other factors might also play a role in the development of cardiac hypertrophy in the reviewed diseases, we discuss the arguments in favor for insulin as a cardiac growth factor in hyperinsulinemic infants with CH.

### Insulin

In the presence of insulin, the insulin receptor (IR) phosphorylates insulin receptor substrate proteins (IRS proteins) that are linked to the activation of two main signaling pathways: the phosphatidylinositol 3-kinase (PI3K)–AKT/protein kinase B (PKB) pathway, which is responsible for most of the metabolic actions of insulin, and the Ras–mitogen-activated protein kinase (MAPK) pathway and regulates expression of some genes to control cell growth and differentiation [85]. Insulin is merely known for its metabolic effects and the heart is an important target for insulin [86–88]. Insulin receptors are present in cardiomyocytes in a density comparable to other insulin sensitive tissues [87]. In humans, insulin responsiveness is established during the last trimester of gestation [89]. In late gestation, there is high fetal susceptibility as a result from higher insulin/IGF-1 receptor expression on fetal cardiomyocytes and failure to downregulate insulin receptors in response to high insulin concentrations [90, 91]. Downstream of the insulin receptor, glycogen synthase kinase-3β negatively regulates cardiac hypertrophy. Since expression of this enzyme is inhibited by insulin, it is a potential mechanism for CH in the hyperinsulinemic fetus.

### IGF-1

The heart is an important target for IGF-1 [86, 88] and IGF-I receptors are present in fetal and adult myocardium [88]. There is clinical and experimental evidence that IGF-I plays a key role in normal cardiac growth. In cultured neonatal rat cardiomyocytes, IGF-I induces cardiac hypertrophy and inhibits cardiomyocyt apoptosis. Mice treated with IV IGF-I and rats treated with SC IGF-I have increased protein levels, mainly localized in the heart [92]. IGF-1 is involved in pre- and postnatal growth [93]. IGF-I is secreted by various organs, mainly the liver, under the influence of growth hormone. Prenatal IGF-I-mediated growth is growth hormone independent, in contrast to postnatal growth [92]. Postnatally, childhood growth hormone deficiency is associated with reduced cardiac mass, which increases upon growth hormone treatment, whereas ventricular hypertrophy is noted in acromegaly.

While IGF-I is clearly a cardiac growth factor, its direct role in the development of CH is unproven. The association between IGFR levels and CH is difficult to establish since IGF plasma levels are not necessarily reflective of ligand available to bind to receptors in the heart. While insulin is made in the pancreatic β cells and acts in an endocrine fashion, IGFs are made throughout the body, especially in the liver, skeletal muscle, and heart, and can act in endocrine and paracrine fashion. Since the concentration of IGF-BP1, one of the six IGF binding proteins to which IGF-I is bound, is influenced by insulin, any statement about the role of IGF-I in hyperinsulinism-induced CH is difficult.

### Insulin and IGF1 receptors

The role of the insulin receptor and the IGF-I receptor in cardiac growth has been investigated in transgenic mice. CIRKO mice with specific cardiac insulin receptor knock out have smaller hearts at birth [94]. Muscle-specific IGF-I receptor knock-out mice appear normal, whereas double knock out mice (lacking the insulin and the IGF-I receptor) have a reduced heart size and early mortality, suggesting that the insulin receptor is more critical for cardiac growth than the IGF-I receptor [94].

### Arguments in favor for insulin as a cardiac growth factor in hyperinsulinemic infants with CH

CH is observed in patients with a broad spectrum of hyperinsulinemic diseases indicating that the cardiac hypertrophy develops regardless of the etiology of the hyperinsulinism. Presumably, the association between hyperinsulinism and CH is underestimated as diagnostic options for CH by echocardiography have improved only in the last decades [95] and since many case reports have incomplete clinical and laboratory information including missing insulin levels. In addition, some patients with clinical evidence of hyperinsulinism do not have very elevated insulin levels. Clinical data emphasize that the severity of hyperinsulinism is the main factor to determine whether CH develops or not and whether CH is symptomatic or not since the clinical course of CH in general parallels the serum insulin levels. In congenital hyperinsulinism, patients that ultimately require pancreatectomy have the highest risk of CH [82]. In patients with milder congenital hyperinsulinism and infants of diabetic mothers, CH is mostly reversible and rarely fatal. In the latter patients, insulin levels are not always very elevated but inappropriately increased in the presence of hypoglycemia [96]. In some patients, insulin levels are often not increased at the time of hypoglycemia, because of the periodic release of insulin, which is missed by measuring a single serum sample [97]. This can be overcome by collecting several samples during hypoglycemia. In any case, in these patients, insulin levels may fail to predict the presence of CH.

Another argument in favor of insulin as a cardiac growth factor is provided by the difference in mechanism, whereby hyperinsulinemia results in CH in unrelated diseases. Insulin is an anabolic hormone, and interaction with the cardiac insulin receptor as observed in congenital hyperinsulinism and in infants of diabetic mothers might explain the large weight and plethoric aspect of these patients. In contrast, neonates and infants with a defective insulin receptor appear emaciated. In infants of diabetic mothers and congenital hyperinsulinism, the CHCG is presumably due to the interaction of insulin with the intact insulin receptor. In insulin resistance syndromes, it is presumed that insulin acts through the IGF-1 receptor and not through the defective insulin receptor [98]. Indeed, several studies showed that insulin activates the IGF-1 receptor at high concentrations of insulin, but not at low concentrations [84, 99, 100]. This was well documented in leprechaunism patients. Insulin binding to human leprechaunism fibroblasts is impaired, but mitogenic response is noted at high supraphysiologic concentrations of insulin and this response can be blocked by an antibody to the IGF-I receptor [100]. In leprechaunism, which is the most severe form of insulin resistance characterized by very high levels of insulin (up to 6000 μU/ml), the CH is very severe and often the cause of death [39]. This is most pronounced in patients with a null mutation of the insulin receptor [101]. In patients with congenital lipodystrophy, it is not fully understood whether the cardiomyopathy is the direct effect of the mutant gene, an effect of lipotoxic substances or the result of hyperinsulinemia. A causal relationship between hyperinsulinemia and CH in patients with BSCL2 mutations is suggested in two patients in whom CH improved or even regressed after correction of the hyperinsulinism [16, 41].

## Implications for diagnostic work-up

The presumed association between hyperinsulinism and CH emphasizes the need to consider hyperinsulinism in the differential diagnosis of HCM. The main differential diagnoses for cardiac hypertrophy due to hyperinsulinism are metabolic, mitochondrial, and storage disorders such as Pompe’s disease, rather than primary idiopathic HCM, since they, like cardiomyopathy caused by hyperinsulinism, are not characterized by radial systolic hypercontractility which is virtually always present in idiopathic HCM [102]. In addition, the pattern of hypertrophy visualized by echocardiography might help in differentiating between the different etiologies as most patients with hyperinsulinemic disease are reported to have septal hypertrophy or left ventricular outflow tract obstruction, while the concentric hypertrophy pattern is a characteristic feature of metabolic and neuromuscular disorders [2]. However, recent studies using more advanced ultrasound devices should confirm the exact hypertrophy patterns in hyperinsulinemic diseases. It is suggested that in hyperinsulinemic states, the hypertrophy is not limited to the septum and also involves other parts of the heart which could not be imaged with previous echocardiographic techniques.

Regular glucose measurement to detect hypoglycemia is already part of the diagnostic work up of the pediatric CH patient and should alert the physician for underlying hyperinsulinemic disease. After establishing a diagnosis of hyperinsulinism, the etiology can be determined by a thorough physical examination for dysmorphic features, evaluation of pregnancy conditions, and appropriate laboratory investigations. The diagnosis can have significant impact on management and etiology-specific survival. Survival outcomes range from very poor as in leprechaunism to good with spontaneous regression of the hypertrophy in infants of diabetic mothers. Congenital hyperinsulinism and insulin resistance syndromes are genetic diseases, and diagnosis is important in terms of genetic counseling.

The other way around, CH should be recognized as a potential co-morbidity in all neonates with known hyperinsulinism. In terms of prevention, echocardiography should be performed not only in all patients with a clinical suspicion of CH, but also in neonates and infants with known hyperinsulinism especially when requiring medication [103].

## Clinical interventions for CH in hyperinsulinemic infants

### Endocrine management

Interventional options depend on the underlying cause of the hyperinsulinism. Diazoxide, glucagon, and the somatostatin analog octreotide can be effective in lowering insulin in patients with congenital hyperinsulinism based on K-ATP mutation. It should be noted that with diazoxide, there is a risk for cardiac dysfunction due to fluid overload [104]. Another concern with the use of diazoxide is the recent observation of pulmonary hypertension after diazoxide use [105], although this might be caused by (the undiagnosed underlying) cardiomyopathy. Sirolimus administration was successful in four infants with severe hyperinsulinemic hypoglycemia, who were unresponsive to diazoxide and octreotide [106]. Data on cardiac function were not available, but since sirolimus (formerly called rapamycin) inhibits the mTor pathway, which is part of two distinct growth promoting serine/threonine complexes in the Akt pathway of the receptor, it could potentially counteract CH. In some cases, CH is resistant to medical therapy and pancreatic surgery is needed to reduce insulin production. Additional therapies for hyperinsulinism were reported on trial basis or in individual cases. In one patient with BSCL2, Metformin treatment, a mitochondrial glycerophosphate dehydrogenase [107], resulted in normalization of cardiac hypertrophy [41]. Recombinant IGF-1 was administered in several patients with leprechaunism [108, 109] and reported to stabilize severe obstructive cardiac hypertrophy in one patient [110]. Important to note is that IGF-1 treatment could potentially aggravate CH, in view of the proposed pathway of CH development through the interaction with the IGF-1 receptors. Interestingly, reoccurrence of mild cardiac hypertrophy was observed during IGF-1 treatment in one patient with lipodystrophy [111]. Careful monitoring is therefore mandatory when IGF-1 is used in this setting. Finally, leptin and its analogue metreleptin improved metabolic parameters in patients with lipodystrophy and Rabson Mendenhall syndrome [87, 112].

### Cardiac management

In most instances of hyperinsulinemic CH, no specific cardiac therapy is required apart from being very vigilant that the patient becomes neither hypoglycemic nor hypovolemic. Some patients with severe hyperinsulinemic CH might need supportive medication during diagnostic work-up. As outlined before, severe hyperinsulinemic CH is characterized by cardiac stiffness with decreased diastolic filling and an obstructive outflow tract, and patients might therefore benefit from B-blockers [9, 113, 114]. The choice of B-blocker should be carefully considered since some non-selective B-blockers (e.g., propranolol) are reported to have adverse effects in young infants leading to hypoglycemia [115].

## Conclusion

In conclusion, we showed that hyperinsulinism is associated with CH in a broad range of hyperinsulinemic diseases and should therefore be considered in the differential diagnosis of HCM. Insulin might act as a cardiac growth factor in these diseases by interaction with cardiac insulin and IGF-1 receptors. However, the direct causal role of insulin in the development of CH in hyperinsulinemic disease still needs to be definitely established and other contributing factors need to be elucidated since CH is not present in all hyperinsulinemic states. However, the clear relationship between hyperinsulinemia and CH has implications for clinical practice as CH should be recognized as a potential co-morbidity in all neonates with known hyperinsulinism. Visa versa, hyperinsulinemic disease should be considered in neonates/young infants presenting with CH. In the case of suspicion of hyperinsulinemic disease, establishment of the correct diagnosis is essential since it may have prognostic and therapeutic consequences.

## Abbreviations

AGPAT: Acylglycerolphosphate acyltransferase
BSCL: Bernadelli Seip complete lipodystrophy
BWS: Beckwith-Wiedemann Syndrome
CDG: Congenital disorders of glycosylation
CHI: Congenital hyperinsulinism
HCM: Hypertrophic cardiomyopathy
HRAS: Harvey rat sarcoma
IGF: Insulin like Growth Factor
INSR: Insulin receptor
Kir: Potassium channel inward-rectifying
PHHI: Persistent hyperinsulinemic hypoglycemia of infancy
PTRF: Polymerase I and transcript release factor
pUPD11p: Paternal uniparental isodisomy for chromosome 11p (pUPD11p)
Sur: Sulfonylurea receptor
